# Progress in Orthopædic Surgery

**Published:** 1902-08-23

**Authors:** 


### Progress in Orthopædic Surgery.

**The Treatment of Paralytic Deformities by Arthrodesis and Tendon Grafting.**—Botherzat [1] contributes an important article on arthrodesis of the shoulder joint as a means of treatment of paralytic dislocation, which is usually forwards and inwards. The writer points out that there are two groups of infantile paralysis about the shoulder joint. In one group paralysis of that joint is associated with a similar lesion of the scapular muscles. In the other the scapular muscles are sound, while the shoulder muscles are atrophied. It is manifest that the first class are not fit cases for surgical treatment; while in the second class much can be done by arthrodesis, for the paralysis may be limited to the deltoid alone, or it may only affect in addition the supra-spinatus, infra-spinatus and teres minor. Sometimes in addition the triceps is also affected. The condition can be benefited either by the use of apparatus or by operation, but the former are complicated and costly; they tend to cause soreness of the skin, and they further limit the action of the neighbouring healthy muscles. A far better result is obtained by ankylosis of the shoulder joint or arthrodesis. The operation is carried out by an anterior incision which is carried a little inside the acromio-clavicular articulation, and is prolonged downwards and forwards in the interval between the pectoralis major and the deltoid. The capsule is opened over the bicipital groove and the coraco-acromial ligaments divided. As much as possible of the synovial membrane is scraped away, and the cartilage is removed from the head of the humerus and from the glenoid cavity, a proceeding which is not very easy to carry out in its entirety. The head of the humerus is then fixed in internal rotation, with the arm at an angle of 45° with the axillary border of the scapula. The head of the humerus is attached to the glenoid cavity by two silver sutures. The wound is stitched up and the arm placed in a plaster of Paris bandage. Gradually the ankylosis becomes solid, and if massage and electricity are applied to the scapular muscles a good result follows. In the writer's three cases the patient was able to carry the hand to the mouth and use the limb for a very large number of purposes.

The treatment of paralytic deformities elsewhere by means of tendon transplantation is the subject of numerous communications. Dr. Royal Whitman [2] discusses the whole subject in a communication to the New York Academy of Medicine and the indications for it. He states that it is mostly applicable in the lower extremity; while it is not often possible in cases of paralysis of the upper extremity. He thinks that the operation is palliative rather than curative, and that it will supplement rather than supplant mechanical treatment. Further communications on the subject were made by Dr. W. R. Townshend [3] and Dr. Virgil P. Gibney,[4] who gave the results of 92 operations. Among the essentials for the operation the latter quotes primary union, avoidance of the pinching of tendons with forceps and very careful handling of them. The reinforcing tendon should be transplanted into the point of attachment of the tendon of the paralysed muscle. The best material is silk, and it has been found that silkworm gut has given rise to fistula. Of the 92 operations 69 have been traced, and of the latter the results were good in 32, fair in 44, and negative in 24 per cent.

An interesting neurological question is involved in tendon transplantation in the fact that a flexor tendon when transplanted into an extensor gave rise to extension, which has caused considerable perturbation of mind among neurologists. Dr. Joseph Collins [5] stated that, in his opinion, the extension was quite simple and was easily understood by an illustration of the starting of a nervous impulse from the nervous centres. A locomotive leaving a station in New York would go either to Boston or Buffalo, according to the track to which it was switched, and the fact that it had been accustomed for many years to go to Boston did not interfere in any way with its going to Buffalo, provided that it was shunted upon the proper track. A. H. Tubby [6] communicated the results of 11 cases of tendon grafting for paralytic talipes and four cases for spastic paralysis affecting the forearm and hand, which had been operated upon by his method as described in the *British Medical Journal*, 1898, and consisting of the transplantation of the tendon of the pronator radii teres, with section of the flexor tendons of the wrist. His results were good, but he holds that tendon grafting is not an operation for the display of anatomical knowledge nor of mechanical skill in operating, but one requiring the nicest care in the selection of cases and of the muscles to be employed, and careful watching of the results of the operation for several years afterwards. Sinclair White [7] records his experience of 11 cases. He on occasion employed a modification of the operation which was introduced by Vulpius, where the usual method was reversed, the distal end of the divided paralysed tendon being displaced and stitched to a normally situated healthy muscle.

The operation may be done for other paralytic conditions, such as traumatic nerve lesions. As an example of this a case shown by Mr. A. H. Tubby and Dr. Purves Stewart [8] under the title of "A New Method of Treating Old-Standing Paralysis of the Upper Root of the Brachial Plexus" (Erb Duchenne type) is very interesting. The case was that of a carman age fifty-seven who, as the result of a fall two years and three months ago on to his left shoulder and left side of the head, had marked paralysis and atrophy about the left shoulder and upper arm with total loss of electrical excitability in the deltoid, infra-spinatus, biceps, brachialis anticus and supinator longus on that side. Twenty months after the accident, with the object of restoring the power of flexion of the elbow, Mr. Tubby transferred part of the triceps and fixed it into the biceps. A month later an attempt was also made to restore power to the deltoid by attaching it to the humeral part of the pectoralis major. The result was great improvement in the movements and utility of the limb, a very useful amount of flexion of the fore-arm being secured.

**Shortened Limbs.**—The evil effects of the shortening of one limb ; pain, bad progression in walking, and the deformities of the back, which frequently follow asymmetry, and the difficulty of finding surgical means to arrest these evil results, have exercised the minds of surgeons for many years, and many attempts have been made to prevent them. Of course the shortening can be treated by means of a cork sole placed under the foot, but this is often cumbrous and unsightly, and begs the question. If, for instance, the shortening be situated in one bone only the effect of the use of a cork sole is that certain joints are not on the same plane. For example, if the femur be shortened, the effect of a cork sole will be to place the two knee-joints in different planes, although the length of the leg is artificially restored. Mr. G. P. Newbolt[9] describes a case of deformity arising from arrested growth in one limb remedied by exsection of bone from the other. He removed from the left femur (the longer one) one and three-quarter inches of bone, and the result was excellent. He gives as his reason for not removing a full amount of bone that he feared the muscles of the thigh might not contract sufficiently, and that the knee-joint would be left in a weak and flail-like condition. He adds that he has seen such a condition occasionally in cases of fracture of the shaft of the femur with much shortening, and he thought that by letting the patient get about fairly early there would be some absorption of the newly-joined ends of bone, and this would diminish the superfluous half.

**Congenital Displacement of the Hip.** — Dr. Llewellyn Phillips[10] describes the changes which have taken place in an adult pelvis as the result of a congenital dislocation of the hip joint. The pelvis was markedly asymetrical, the left half, that is on the affected side, being less developed. The left ilium was smaller and more vertical. The brim was markedly oblique, and the symphysis lay opposite the right anterior sacral foramina. The curve of the brim was less on the left side than on the right, and the transverse diameter was much diminished While on the subject of congenital hip displacement, we may note a communication from Mr. H. A. Reeves.[11] Mr. Reeves is one of those who still hold the view that no known operation can remedy this malformation. He had seen three cases after operation which failed to convince him of its usefulness, and he alludes to deaths which have occurred as the result of the operation. Mr. Reeves has for years treated his cases in the following way ; in fact, it seems that precedence should be given to him rather than to Lorenz. Mr. Reeves reduces the femoral head into the deficient acetabulum by the methods of Bigelow and Paci combined, and while the limb is kept in position he puts on his extension instrument, which is very simple, and much like a knee caliper splint with a pelvic band. Those who have seen Mr Reeves's cases are satisfied that his method gives a. good results, and better than many others which have been attempted. The fact that Lorenz has abandoned the open operation, and has adopted what he calls the bloodless rectification of congenital dislocations, speaks very much in favour of Mr. Reeves's almost lifelong contention that these cases are, so far as our present knowledge goes, best treated by reduction of the head of the femur into the best position possible and the maintenance of that position by apparatus.

[1] Revue de Chirurgie, May 10, June 10, July 10, 1901. [2] New York Med. Rec., March 29, 1902, p. 516. [3] Ibid. [4] Ibid. [5] Ibid. [6] Pediatrics, 1901, vol. xii., p. 288. [7] Ibid. [8] Brit. Med. Jour., Feb. 1, 1902. [9] Lancet, Sept. 1, 1901. [10] Brit. Med. Jour., Dec. 7, 1901. [11] Lancet, Nov. 23, 1901, p. 1408.

